# Disseminated *Mycobacterium avium* Complex Infection Following CD3/CD20 Bispecific Antibody Therapy in a Patient With Follicular Lymphoma

**DOI:** 10.1093/ofid/ofae460

**Published:** 2024-08-08

**Authors:** Jessica S Little, Rocio M Hurtado, Nicholas Boire, Lindsey R Baden, Alvaro C Laga, Ann W Silk, Caron A Jacobson

**Affiliations:** Division of Infectious Diseases, Brigham and Women's Hospital, Boston, Massachusetts, USA; Harvard Medical School, Boston, Massachusetts, USA; Stem Cell Transplant and Cellular Therapy, Dana-Farber Cancer Institute, Boston, Massachusetts, USA; Harvard Medical School, Boston, Massachusetts, USA; Division of Infectious Diseases, Massachusetts General Hospital, Boston, Massachusetts, USA; Harvard Medical School, Boston, Massachusetts, USA; Division of Pathology, Brigham and Women's Hospital, Boston, Massachusetts, USA; Division of Infectious Diseases, Brigham and Women's Hospital, Boston, Massachusetts, USA; Harvard Medical School, Boston, Massachusetts, USA; Stem Cell Transplant and Cellular Therapy, Dana-Farber Cancer Institute, Boston, Massachusetts, USA; Harvard Medical School, Boston, Massachusetts, USA; Division of Pathology, Brigham and Women's Hospital, Boston, Massachusetts, USA; Harvard Medical School, Boston, Massachusetts, USA; Department of Medical Oncology, Dana-Farber Cancer Institute, Boston, Massachusetts, USA; Harvard Medical School, Boston, Massachusetts, USA; Stem Cell Transplant and Cellular Therapy, Dana-Farber Cancer Institute, Boston, Massachusetts, USA

**Keywords:** disseminated *Mycobacterium avium* complex infection, bispecific antibody therapy, opportunistic infection, immunotherapy

## Abstract

Infections remain a major concern following bispecific antibody therapy but are not well described in pivotal trials. We present the first well-documented case of a classic but rare opportunistic infection, disseminated *Mycobacterium avium* complex, in a patient receiving bispecific antibody therapy.

Despite the rapid expansion of newly approved, highly immunosuppressive oncologic therapies in recent years, including small molecules, monoclonal and bispecific antibodies (BsAbs), and cellular therapies, infection reporting in pivotal clinical trials of these agents remains inadequate [1]. Most importantly, reporting of key opportunistic infections (OIs) is inconsistent, and many infections are characterized only by syndrome rather than by pathogen, limiting conclusions about optimal prevention and monitoring strategies [1]. The lack of clear definitions around what constitutes an OI also may lead to heterogeneity of reporting. Thus the emergence of rare and unexpected OIs in patients receiving these novel therapies postmarketing is of critical importance. BsAbs or bispecific T-cell engagers are among these newly approved immunotherapies for patients with relapsed/refractory malignancies, and act by binding 2 epitopes to link tumor cells to effector T cells, triggering tumor lysis [2]. BsAb therapy has been approved for treatment of multiple myeloma (MM), acute B-cell lymphoblastic leukemia, and B-cell lymphomas including 3 CD20xCD3 bispecific antibody therapies approved for relapsed/refractory B-cell lymphomas since 2022 (Supplementary Table 1) [3]. Despite their therapeutic promise, these agents are associated with significant toxicities including cytokine release syndrome, immune effector cell–associated neurotoxicity syndrome, and infections [2, 4]. However, although an increased risk of infections including OIs has been highlighted following BsAb therapy for MM, the infection risk among patients with lymphoma is not well understood [5–7]. Here, we report for the first time a well-documented case of a “classic” OI following treatment with CD3/CD20 BsAb therapy.

## CASE DESCRIPTION

A 75-year-old man with remote history of thyroid cancer treated with thyroidectomy and radioactive iodine in 2001 and relapsed/refractory follicular lymphoma treated with experimental CD20xCD3 BsAb therapy (odronextamab), presented with new bony lesions and lymphadenopathy on surveillance positron-emission computed tomography scan (PET/CT).

The patient was diagnosed with follicular lymphoma in 2007 and received multiple lines of therapy including rituximab, cyclophosphamide, vincristine, prednisone; bendamustine; idelalisib; and CD19 CAR T-cell therapy (axicabtagene ciloleucel; June 2018) with second infusion in September 2019 without significant infectious complications. He developed progressive disease and was initiated on a CD20xCD3 BsAb (odronextamab) in October 2020 via clinical trial without cytokine release syndrome or neurotoxicity requiring corticosteroid therapy. In January 2021, he had evidence of complete remission by bone marrow biopsy and by PET/CT with no other abnormalities noted; he continued maintenance BsAb therapy. In March 2023, BsAb therapy was stopped because of fatigue, low CD4 T-cell counts, and recurrent advanced squamous cell carcinomas of the scalp with multiple in-transit metastases. In terms of the CD4 lymphopenia, the patient had a low CD4 count of 82 cells/µL in June 2020, 9 months after CAR T-cell therapy and before disease relapse, however following initiation of CD20/CD3 BsAb, CD4 T-cell count dropped to a nadir of 5 cells/µL (Supplementary Figure 1).

**Figure 1.**
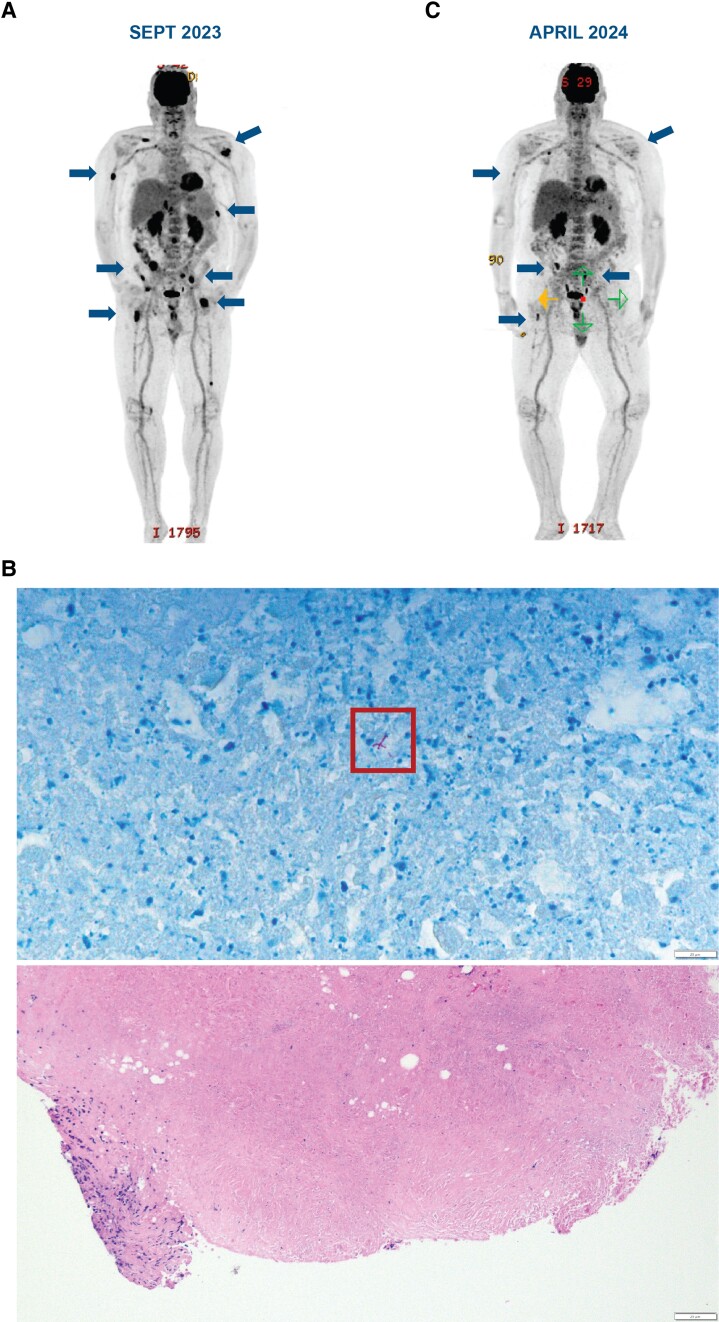
Positron emission tomography/computed tomography prior to treatment (A) and following treatment (C) and histopathologic findings (B) of disseminated *Mycobacterium avium* complex infection in a patient following bispecific antibody therapy.

One month after stopping BsAb therapy, surveillance PET/CT demonstrated multiple new areas of fluorodeoxyglucose (FDG) uptake in the bone marrow including the bilateral humeri, iliac bones, and femurs, and left posterolateral 10th rib (Figure 1*A*). At the time, the patient reported fatigue, but no fevers/sweats, weight loss, or bone pain. He underwent CT-guided biopsy of the right iliac bone in April 2023, which was nondiagnostic and interpreted as focal spindle cell proliferation without malignancy. Bone marrow biopsy also showed no evidence of malignancy. Repeat PET/CTs in June and September showed increased size and FDG uptake of the bony lesions as well as new FDG-avid periportal and retroperitoneal lymph nodes. A second CT-guided biopsy of the right iliac bone in October 2023 demonstrated spindle cell proliferation, granulomatous inflammation, and focal necrosis. No bacteria, fungi, or mycobacteria were detected on special stains. Neither of the initial bone biopsies was sent for microbiologic culture.

**Figure 2.**
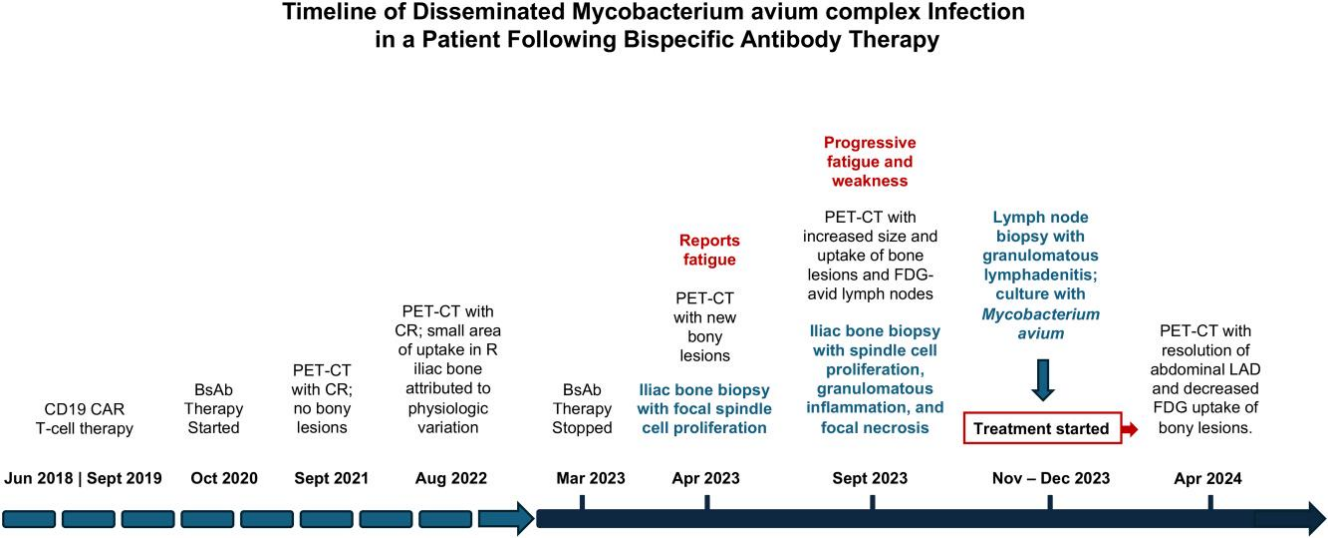
Timeline of disseminated *Mycobacterium avium* complex infection in a patient following bispecific antibody therapy.

The patient was evaluated in an infectious diseases clinic in November 2023 where he reported increased fatigue, generalized weakness, and headaches. He denied fevers, sweats, weight loss, respiratory symptoms, abdominal pain, diarrhea, bone/joint pain, or rash. He had lived in New England throughout his life and worked previously as a carpenter with extensive exposure to dust and soil. He reported prior travel to San Diego, Arizona, Italy, Costa Rica, and Quebec on short vacations and denied recent notable water or animal exposures and reported no new sexual contacts or use of illicit substances. He had no structural lung disease or abnormal pulmonary findings on PET/CT. Laboratory testing was notable for normal renal/liver function and mild leukopenia with white blood cell count of 2.68 K/µL with 62% neutrophils, 14% lymphocytes, 17% monocytes, and 3% bands. CD4 T-cell count was 43 cells/µL and immunoglobulin G level was 307 mg/dL. Broad microbiologic work up was negative, including HIV 1/2 antibody/antigen test, 1,3-β-d-glucan, galactomannan antigen, histoplasma serum/urine antigens, histoplasma serum antibody, Blastomyces urine antigen, coccidioides urine antigen and serum antibody, brucella antibody, bartonella antibody, and polymerase chain reaction testing. A metagenomic cell-free DNA test for pathogens (Karius; detects >1000 bacteria, fungi, parasites, and viruses) was negative. An interferon gamma release assay was not analyzed because of insufficient cells. Mycobacterial blood cultures were ordered but not performed because of a laboratory error; a mycobacterial urine culture was negative. The patient underwent a laparoscopic lymph node biopsy in November 2023 that revealed necrotizing granulomatous lymphadenitis with acid-fast bacilli best visualized on Fite stain, consistent with mycobacterial infection (Figure 1*B*). Acid-fast bacilli smear was negative, but *Mycobacterium avium* was isolated in tissue culture in mid-December. The patient was initiated on therapy with azithromycin, ethambutol, and rifabutin for disseminated *Mycobacterium avium* complex infection. His treatment course was complicated by neutropenia attributed to rifabutin, requiring a switch to rifampin that was well tolerated, and progressive squamous cell carcinoma of the scalp requiring concomitant initiation of immunotherapy. Repeat PET/CT in April 2024 showed resolution of the prior FDG-avid intraabdominal lymph nodes and resolution of most focal areas of bony uptake with several small residual areas of infection (Figure 1*C*). Repeat CD4 T-cell count in April 2024 remained low at 39 cells/µL. The full clinical course is outlined in Figure 2.

## DISCUSSION

Here, we describe the first case of disseminated *Mycobacterium avium* infection (MAC) in a patient who received BsAb therapy. Disseminated MAC infections are exceedingly rare, primarily reported in the context of advanced HIV/AIDS or primary immunodeficiencies, and have uncommonly been reported in patients with hematologic malignancy or even following hematopoietic cell transplantation, despite substantial associated cell-mediated immunodeficiencies [8, 9]. Disseminated MAC infections may present with vague symptoms including fatigue, weight loss, fever, night sweats, or diarrhea, though our patient presented only with fatigue and weakness, making the diagnosis more challenging [9]. Microbiologic diagnosis can be difficult, and notably in this patient, a laparoscopic lymph node biopsy was required to make a diagnosis. Two prior bone biopsies were not sent for microbiologic culture highlighting the importance of obtaining microbiologic and histopathologic evaluation. Treatment for disseminated MAC infection is typically characterized by a prolonged course of multiple antibiotics, with significant associated adverse effects and drug-drug interactions that may present challenges for any ongoing chemoimmunotherapy [10].

Opportunistic infections have been reported in the setting of BsAb therapy including cytomegalovirus viremia and disease, Epstein-Barr virus infection, serious adenovirus infections, toxoplasmosis, and *Pneumocystis jirovecii* pneumonia, though detailed descriptions of these events are often limited [6, 7, 11, 12]. The emergence of this rare OI, seen most often in patients with advanced HIV/AIDS, has important implications for the long-term monitoring of patients receiving BsAb therapy. This patient showed evidence of T-cell immunodeficiency (<100 cells/µL) before initiation of BsAb that worsened and persisted following BsAb treatment with a CD4 T-cell count <50 cells/µL more than 1 year after cessation of BsAb therapy, highlighting the potential impact of T-cell exhaustion after long-term BsAb with consequences including OIs [13]. In particular, this case raises the question of whether extended durations of these therapies (>2 years) may have a cumulative impact on infection risk—a factor that may not be fully reflected in pivotal trials with shorter durations of follow up. Preceding therapies such as CAR T-cell therapy (>3 years prior) may have also had an impact given his low baseline CD4 T-cell count and could have implications for the sequencing of these immunosuppressive therapies, though no cases of disseminated MAC related to CAR T-cell therapy have been reported to our knowledge.

Finally, this case illustrates the challenges in diagnosing these rare OIs, with 2 negative bone biopsies, broad negative microbiologic evaluation, and progression of symptoms over 7 months, emphasizing the importance of maintaining a high index of suspicion for infection in highly immunocompromised patients, including those receiving BsAbs. Early involvement of infectious diseases teams in the care of patients on BsAb therapy with unusual presenting symptoms may impact time to diagnosis. Improved reporting of OIs in clinical trials and real-world studies with emphasis on the cumulative risk of infection with continuous BsAb therapy will be vital to better characterize optimal monitoring and preventive strategies [1]. Current guidance on antimicrobial prophylaxis remains limited for BsAb therapy and is primarily based on expert opinion, though herpes simplex virus/varicella zoster virus antiviral prophylaxis and *Pneumocystis jirovecii* prophylaxis are generally used for BsAb recipients with MM and B-cell lymphomas [2]. The benefit of any additional prophylaxis for patients with CD4 lymphopenia remains unclear and additional studies are needed to inform management of these highly immunosuppressed patients.

## Supplementary Material

ofae460_Supplementary_Data

## Supplementary Data

Supplementary materials are available at *Open Forum Infectious Diseases* online. Consisting of data provided by the authors to benefit the reader, the posted materials are not copyedited and are the sole responsibility of the authors, so questions or comments should be addressed to the corresponding author.

## Notes

***Authorship contributions***. J.S.L. wrote the manuscript and created the figures. N.B. assisted in the case, provided the photos of the pathologic findings, and edited the manuscript. R.H., A.L., and A.W.S. assisted in the case and edited the manuscript. C.J. assisted in the case and helped to conceptualize and edit the manuscript.

***Patient consent statement***. The patient's written consent was obtained and identifying information was removed from the manuscript. Procedures were performed in accordance with the Helsinki declaration.

***Financial support*.** No funding was used for this study.
