# Supplementary material for: Disseminated Mycobacterium avium Complex Infection Following CD3/CD20 Bispecific Antibody Therapy in a Patient With Follicular Lymphoma
Source: Open Forum Infect Dis. 2024 Aug 8;11(9):ofae460. doi: 10.1093/ofid/ofae460 (PMC11368541; doi:10.1093/ofid/ofae460)
Supplement: ofae460_Supplementary_Data [file ofae460_supplementary_data.zip › Disseminated MAC BsAb Supplemental 07_15_24 Tracked.docx]

**Disseminated *Mycobacterium avium complex* Infection following CD3/CD20 Bispecific Antibody Therapy in a Patient with Follicular Lymphoma**

***Supplemental Materials***

**Table S1. Approved Bispecific Antibody Therapies for Hematologic Malignancies**

| Indication | Agent | Target | Year |
| --- | --- | --- | --- |
| B-cell Acute Lymphoblastic Leukemia (B-ALL) | Blinatumomab | CD3/CD19 | 2014 |
| Multiple Myeloma | Teclistimab | CD3/BCMA | 2022 |
|  | Elranatamab | CD3/BCMA | 2023 |
|  | Talquetamab | CD3/GPRC5D | 2023 |
| B-cell Lymphomas | Mosunetuzumab | CD3/CD20 | 2022 |
|  | Glofitamab | CD3/CD20 | 2023 |
|  | Epcoritamab | CD3/CD20 | 2023 |

**Figure S1. CD4 T Cell counts Prior to and Following Bispecific Antibody Therapy in a Patient with Subsequent Disseminated Mycobacterium avium complex Infection**

**
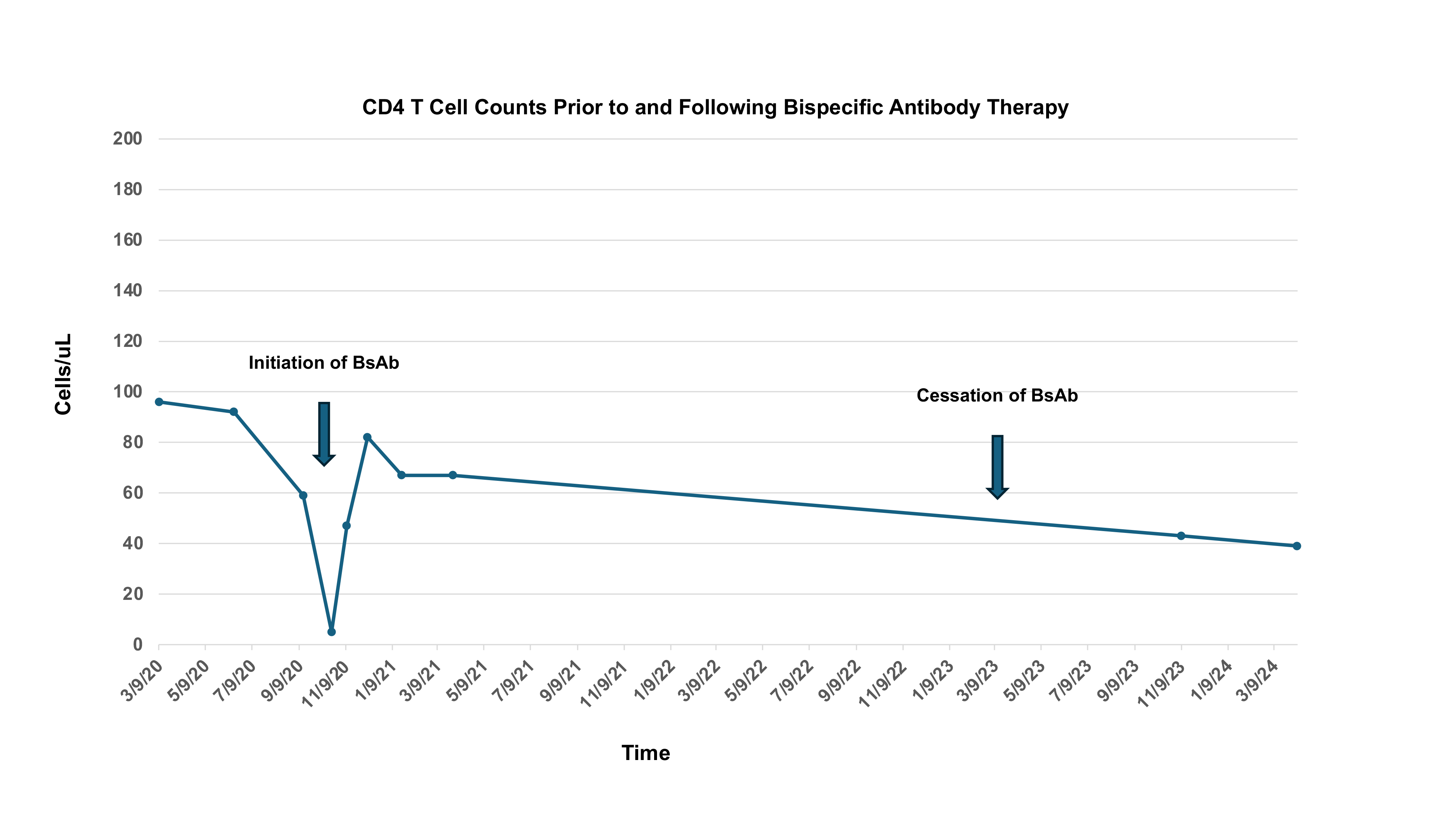
**
