# Supplementary figures and images for: Disseminated Mycobacterium avium Complex Infection Following CD3/CD20 Bispecific Antibody Therapy in a Patient With Follicular Lymphoma
Source: Open Forum Infect Dis. 2024 Aug 8;11(9):ofae460. doi: 10.1093/ofid/ofae460 (PMC11368541; doi:10.1093/ofid/ofae460)

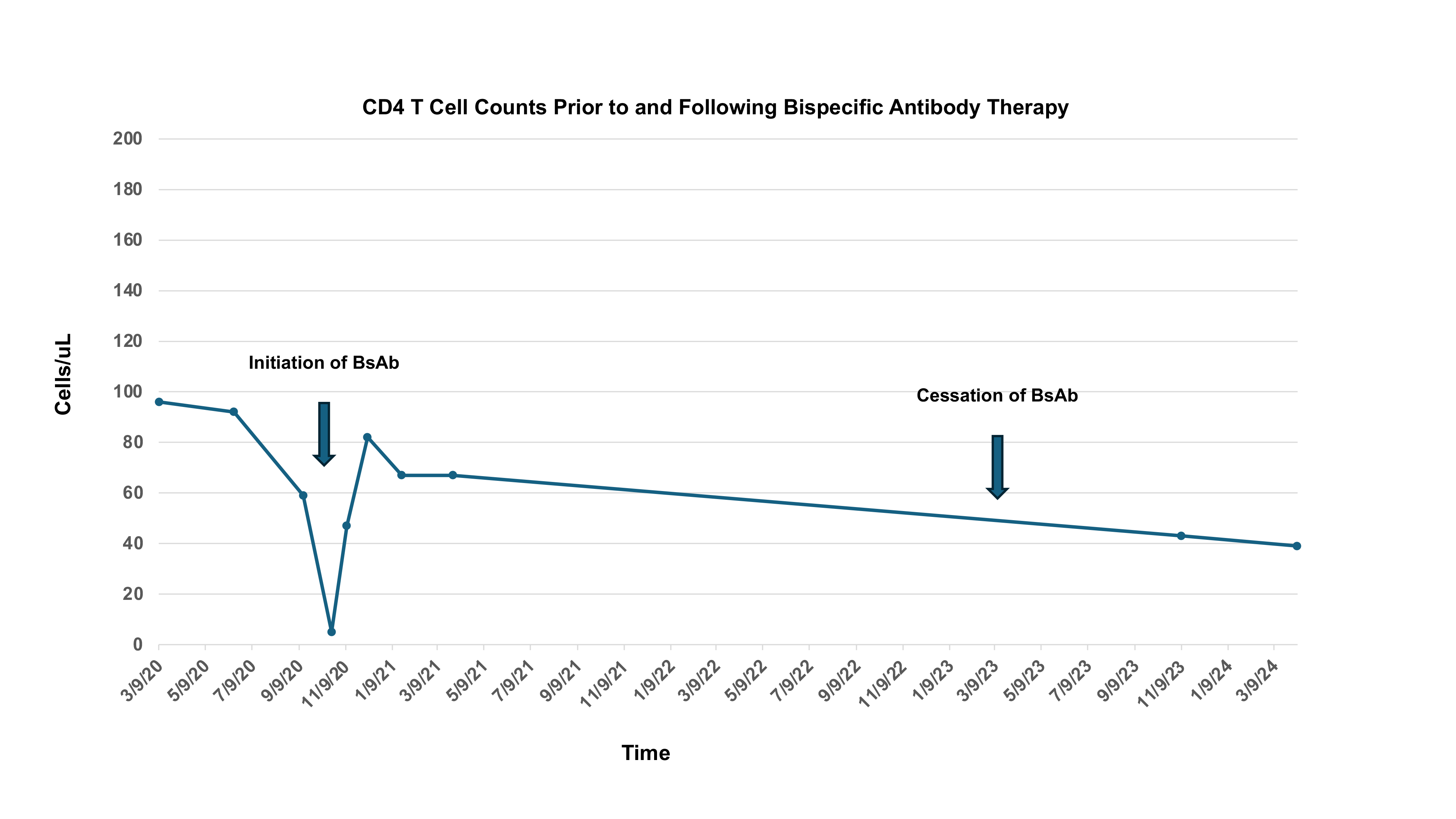

Supplement: ofae460_Supplementary_Data [file ofae460_supplementary_data.zip › Supp Fig 1 Lymphoma BsAb.png]
